# Moringa leaf extract and green algae improve the growth and physiological attributes of Mentha species under salt stress

**DOI:** 10.1038/s41598-022-18481-5

**Published:** 2022-08-20

**Authors:** Wafa’a A. Al-Taisan, Nadiyah M. Alabdallah, Lolwah Almuqadam

**Affiliations:** 1 Department of Biology, College of Science, Imam Abdulrahman Bin Faisal University, 1982, Dammam, 31441 Saudi Arabia; 2 Basic and Applied Scientific Research Center (BASRC), Imam Abdulrahman Bin Faisal University, P.O. Box 1982, Dammam, 31441 Saudi Arabia

**Keywords:** Biochemistry, Plant sciences

## Abstract

Climate change, food scarcity, salt stress, and a rapidly growing population are just a few of the major global challenges. The current study examined into whether *Moringa oleifera* (L.) leaf extract and green algae (*Ulva intestinalis*) could help improve salt tolerance in *Mentha* species (*Mentha piperita; Mentha longifolia*). *Moringa* leaf extract (MLE) and green algae (GA) were applied to *Mentha* seedlings under three different salt treatments: 0 mM, 20 mM, 40 mM, 60 mM, and 90 mM, respectively. For each treatment, three biological replicates were conducted, with each replicate containing at least three plants. *Mentha* species were negatively affected by salt stress in terms of shoot length, fresh and dry weight, photosynthetic pigments, and antioxidant enzyme activities. However, the use of MLE and GA significantly improved the development and physiology of *Mentha* species under salt stress conditions. The MLE and GA treatments dramatically (*p* ≤ 0.001) increased SOD activity by 7% and 10%, CAT activity by 16% and 30%, APX activity by 34% and 56%, GPX activity by 12% and 47%, respectively, in *Mentha piperita* seedlings, which in turn strikingly increased superoxide dismutase (SOD) activity by 6% and 9%, catalase (CAT) activity by 15%, 28% and 44%, 27%, ascorbate peroxidase (APX) activity by 39% and 60%, glutathione peroxidase (GPX) activity by 23% and 58%, respectively, in *Mentha longifolia* seedlings, relative to the control. Aiming to answer questions about the relationship between plant extraction and traditional agricultural methods, this research greatly advances the goal of sustainable development for improving plant productivity by providing a much safer and more environmentally friendly adaptability.

## Introduction

The effects of climate change are having a negative impact on agricultural production all around the world^1–3^. Salinization of the soil due to climate change is causing yield loss worldwide. Salt stress is one of the most important abiotic stresses limiting plant development and productivity, especially in arid and semi-arid regions around the world^4,5^. It has become a significant concern in areas covering around 1125 million hectares worldwide, of which 76 million hectares are directly affected by human activities, resulting in a 1.5-million-hectare annual loss of arable land due to salinization^6^. Exposure to salt stress has dramatically influenced physiological responses such as altered plasma membrane integrity, increased reactive oxygen species (ROS) generation, decreased photosynthetic efficiency, decreased stomatal aperture size, and insufficient accessibility to antioxidant enzymes^7–9^. Furthermore, ROS buildup generates oxidative bursts in cellular compartments, causing proteins, DNA, and lipids to change^10–12^.

Different techniques have been proposed to mitigate the negative effects of salt stress. These approaches include the use of salt-tolerant varieties, stress signaling molecules, osmoprotectants, green algae and plant extracts. Plant and green algae extract, which are both physiologically safe and economically sustainable, have demonstrated a great deal of promise for crop enhancement in moderate-stress conditions in recent years^13^. Water extracts from a variety of cultivated plants have been noted that enhance plant growth and yield in both normal and stressful conditions by altering phytohormone metabolism, photosynthetic activity, and antioxidant defense system^14^. *Moringa oliefera* (L.) has received much interest from researchers because its leaves contain more minerals, growth hormones, vitamins, and antioxidants^15–19^. MLE applied to plant leaves has been demonstrated to promote seedling establishment, seedling growth, and eventually production in abiotically stressed field crops^20–25^. *Ulva intestinalis* L. is a marine green alga in the Ulvaceae family with a tubular frond and unbranched thalli^26^. It is a rich source of physiologically active molecules such as essential fatty acids, vitamins, amino acids, minerals, and growth stimulating substances; they have also been found to boost plant growth performance, antioxidant activities, and tolerance to abiotic stress^27,28^.

*Mentha* species are members of the Lamiaceae family, which possesses medicinal and fragrant properties. Since this particular species displays significant biological activities, it has been utilized as a treatment for a variety of respiratory conditions, including bronchitis, sinusitis, and even the common cold^29^. Moreover, it has the potential to be employed in the pharmaceutical and food industries as an efficient and cost-effective source of natural commercial antioxidants^29^. However, no research has been undertaken to our knowledge on the influence of MLE and GA extracts on the growth and physiology of *Mentha* species under salt stress conditions. Thus, the primary goal of this study is to investigate into the potential effects of MLE and GA on the growth and physiological attributes of *Mentha* species grown under salt stress conditions. The findings of this study will aid in improving *Mentha* species productivity in salt-stressed conditions.

## Materials and methods

### Experimental particulars

The Department of Biology, College of Science, Imam Abdulrahman Bin Faisal University (26.3928° N, 50.1926° E) undertook this study to investigate the effect of MLE and GA on the growth and physiology of *Mentha* species (*Mentha piperita* L. and *Mentha longifolia* L.) identified by Šarić-Kundalić et al.^30^, and growing under salt stress. Cultivated (*Ulva intestinalis* L.) identified based on Budd^31^ techniques, and collected from Az Zakhnuniyah is an island located on the western coast of the Arabian Gulf (N 25° 54′ 72.94″, E 50° 32′ 53.31″) and Moringa (*Moringa oliefera* L.) leaves were collected from Al-Ahsa city market (Voucher number-IAU:104598). On the other hand, *Mentha* seeds were collected from the local market in Dammam, Saudi Arabia. The experiment used a completely randomised design with split plot layouts. Pots (40 cm in height and 25 cm diameter) were filled with compost, sand (45.29%), silt (36.22%), and clay (21.14%), with pH and EC of 7.6 and 2.52 dS m^−1^, respectively. Soil pH was measured by pH meter (Divinext 3), whereas the EC was measured by EC meter (HI98331). In each of the pots, three seeds of each *Mentha* species were sowed. This study was performed with the local (Saudi Arabia) regulations implemented for studying towards the plants.

### Salt stress treatments and preparation of extracts

Treatments were prepared based on the methods of Gholamnia et al.^8^. During the experiment, different doses of NaCl (0, 20, 40, 60, and 90 mM) were added to the experimental pots to produce salt stress. *Moringa* leaves that were mature and healthy were harvested and cleaned with tap water before being stored in the refrigerator overnight. An assembled machine was used for the extraction procedure. Distilled water was used to dilute the extracts to a concentration of 3%. To eliminate pollutants, tap water and distilled water were used to rinse *Ulva intestinalis*. It was homogenized in distilled water (1:1 by volume) at room temperature and stored until further use was needed. 100% of the liquid extract was consumed. The final extract yielded a 2% solution in distilled water.

### Determination of growth parameters

Plant lengths determined by using a metric scale and expressed in centimeter (cm). The plant materials were split into shoots and roots after being cleaned with double distilled water to eliminate sand particles. The fresh weights (FW) and dry weights (DW) were measured with an analytical balance (HR-200) and expressed in grams (g).

### Photosynthetic pigment determination

Arnon^32^ approach was used to extract photosynthetic pigments. At room temperature, a 0.25 g leaf sample was taken and ground with 5 ml of 80% acetone. After that, the extract was centrifuged at 3000 rpm for 10 min at 40 °C. The absorbance of the supernatant at 663 and 645 nm was used to determine the chlorophyll a and b concentrations.

### Proline determination

The Bates et al.^33^ method was used to estimate proline concentration. 10 mL of aqueous sulfosalicylic acid and 0.5 g of newly plucked leaves (3%). After that, the mixture was filtered through a Whatman No. 40 filter paper. The mixture was placed in test tubes, and 2 mL of ninhydrin solution and 2 mL of glacial acetic acid were added. The mixture was then heated at 95 °C for over an hour before being placed in an ice bath to cool. The mixture was then extracted with 10 mL of toluene as a chromophore, and the reaction mixture was constantly circulated via an air stream for 1–2 min to separate the aqueous phase from the chromophore, which contained toluene. Finally, the separated colored phase was allowed to dry at room temperature for 2–3 min before its absorbance was measured with a spectrophotometer to be 520 nm.

### Total sugar content determination

The method described by Du Bois et al.^34^ was used to calculate the total soluble sugar content (1956). To extract 0.5 g of fresh leaves, 10 mL of ethanol (80%) was employed. After centrifugation, the supernatant was combined with 2.5 mL of 5% phenol solution (v/v) and 0.5 mL of sulfuric acid. To heat the combination, it was immersed in a water bath for 20 min. A standard curve was used to calculate the total soluble sugar concentration, and the absorbance at 490 nm was calculated.

### Extraction and measurement of antioxidant enzyme activity

The antioxidant enzymes were extracted using the Mukherjee and Choudhuri^35^ approach. In 10 mL of phosphate buffer, 0.5 g of fresh leaves were extracted (pH 7). After that, the homogenate was centrifuged at 15,000 rpm for 10 min at 4 °C. The supernatant was then maintained at 20 °C to assess antioxidant enzyme activity.

### Superoxide dismutase (SOD) activity determination

The nitro-blue-tetrazolium (NBT) reduction procedure was used to measure SOD activity^36^. The reaction mixture (3 mL) includes 50 enzyme extract, 150 riboflavin (13 M), 2.5 mL methionine (13 M), 250 NBT (63 M), and 50 phosphate buffers (50 mM, pH 7.8). The absorbance at 560 nm was measured using a spectrophotometer (LKB-Biochrom 4050).

### Catalase (CAT) activity determination

The Aebi^37^ approach was used to measure CAT activity. The enzyme extract (40 mL) was combined with 0.016 mL of H_2_O_2_ (30%) and a 10 mM phosphate buffer solution (pH 7.0). Finally, the absorbance at 240 nm was evaluated using a spectrophotometer (LKB-Biochrom 4050).

### Ascorbate peroxidase (APX) activity determination

The APX activity was evaluated using the Nakano and Asada^38^ approach. The reaction mixture contained 0.1 M potassium phosphate buffer (pH 7.0), 0.5 mM ascorbate, 0.1 mM EDTA, 1.0 mM H_2_O_2_, and 20 µL enzyme extract (2.22 mL). The enzyme coefficient of 2.8 mM^−1^ cm^−1^ was used to calculate APX activity.

### Guaiacol peroxidase (GPX) activity determination

The GPX activity was measured at 25 °C using the Elia et al.^39^ technique. The reaction mixture includes 0.2 mL enzyme extract, 10 mM sodium phosphate buffer (pH 7.0), 1 mL H_2_O_2_ (30%), 1 mL guaiacol (0.05 M), and 2 mL distilled water. Guaiacol oxidation was determined by measuring the rise in absorbance at 470 nm over a one-minute period. One unit of POD is the amount of enzyme required to catalyse the reduction of 1 M of H_2_O_2_, using guaiacol as the hydrogen donor, per minute under certain conditions, and was calculated using the enzyme coefficient 26.6 mM^−1^ cm^−1^.

### Statistical evaluation

The MINITAB-17 statistical software was used to perform analysis of variance (ANOVA) on the data, and the results were displayed as treatment mean ± SE (n = 3). The LSD test reveals that bars with the same letter are not statistically different at the *p* < 0.05 level.

## Results

### Growth conditions

To analyze the beneficial effects of MLE and GA on Mentha species, we looked at a variety of morphological traits, including shoot length, shoot fresh and dry weight, and root fresh and dry weight. Shoot length, fresh and dry weight, and root fresh and dry weight were all reduced significantly (p ≤ 0.001) when the *Mentha* species were subjected to varied doses of NaCl (20, 40, 60, and 90 mM) compared to control (Table 1). The salt treatments (20, 40, 60, 90 mM) dramatically (*p* ≤ 0.001) decreased shoot length by 5%, 20%, 29%, 39%, root length by 8%, 20%, 29%, 39%, shoot fresh weight by 32%, 35%, 45%, 70%, shoot dry weight by 7%, 27%, 49%, 64%, root fresh weight by 20%, 37%, 61%, 69%, respectively, which in turn strikingly decreased the root dry weight by 33%, 63%, 73%, 92% in *Mentha piperita* seedlings, relative to the control. Conversely, salt treatments reduced shoot length, fresh and dry weight, and root fresh and dry weight of *Mentha longifolia*. Nonetheless, exogenous MLE and GA treatment significantly improved these parameters in both *Mentha* species when exposed to salt.

**Table 1:** Effect of MLE (*Moringa oleifera*) and GA (*Ulva intestinalis*) on growth parameters in the *Mentha* species seedlings under salt stress (0 mM,20 mM, 40 mM,60 mM, 90 mM).

Species	Growth parameters	Treatment	Salt Concentrations				
			0 mM	20 mM	40 mM	60 mM	90 mM
*Mentha piperita*	SL	Control	24.3 ± 2.4a	23.0 ± 2.12b	19.4 ± 1.9c	17.3 ± 1.8d	15.0 ± 1.7e
		MLE	26.5 ± 1.3a	25.9 ± 1.1b	23.7 ± 0.92c	20.9 ± 0.83d	16.8 ± 0.79e
		GA	29.5 ± 2.9a	28.0 ± 2.4b	27.8 ± 2.2c	23.4 ± 2d	18.7 ± 1.37e
	RL	Control	22.2 ± 1.2a	20.7 ± 1.1b	18.0 ± 1.1c	16 ± 0.92d	13.7 ± 0.81e
		MLE	24.9 ± 1.52a	22.6 ± 1.4b	21.7 ± 1.32c	18.6 ± 1.13d	16.0 ± 1.11e
		GA	28.0 ± 1.6a	26 ± 1.42b	25.1 ± 1.21c	21.6 ± 1.12d	18.1 ± 0.98e
	SFW	Control	3.6 ± 0.23a	2.4 ± 0.19b	2.3 ± 0.18b	1.9 ± 0.09c	1.1 ± 0.08d
		MLE	4.9 ± 0.24a	4.6 ± 0.22b	4.4 ± 0.21c	4.3 ± 0.21c	2.7 ± 0.16d
		GA	7.2 ± 0.46a	6.6 ± 0.43b	6.2 ± 0.38c	5.8 ± 0.37d	4.0 ± 0.28e
	SDW	Control	2.4 ± 0.18a	1.8 ± 0.15b	1.6 ± 0.14c	1.4 ± 0.12c	0.9 ± 0.08d
		MLE	4.0 ± 0.28a	3.5 ± 0.23b	2.9 ± 0.2c	2.2 ± 0.18d	1.5 ± 0.17e
		GA	5.8 ± 0.34a	5.0 ± 0.33b	4.3 ± 0.32c	3.9 ± 0.3d	2.5 ± 0.27e
	RFW	Control	2.8 ± 0.18a	2.3 ± 0.16b	1.8 ± 0.15c	1.1 ± 0.13d	0.9 ± 0.07d
		MLE	5 ± 0.26a	4.2 ± 0.24b	4.2 ± 0.23b	3.9 ± 0.22c	2.3 ± 0.16d
		GA	6.9 ± 0.48a	5.8 ± 0.44b	5.3 ± 0.39bc	4.9 ± 0.33c	3.8 ± 0.3d
	RDW	Control	1.7 ± 0.07a	1.2 ± 0.06b	0.6 ± 0.05c	0.5 ± 0.05d	0.1 ± 0.02e
		MLE	3.5 ± 0.22a	3.0 ± 0.2b	2.9 ± 0.19c	2.2 ± 0.18d	1.4 ± 0.17e
		GA	4.5 ± 0.48a	4 ± 0.42b	3.9 ± 0.38b	2.8 ± 0.37c	1.8 ± 0.24d
*Mentha longifolia*	SL	Control	23.4 ± 2a	22.3 ± 1.8b	19.1 ± 1.7c	16.5 ± 1.4d	10.7 ± 1.2e
		MLE	25.7 ± 2.1a	23.9 ± 2b	22.1 ± 1.67c	18.1 ± 1.54d	15.5 ± 1.34e
		GA	29.1 ± 2.6a	27.1 ± 2.4b	25.9 ± 2c	20.2 ± 1.8d	17.8 ± 1.3e
	RL	Control	21.9 ± 1.38a	20.3 ± 1.2b	16.9 ± 1.18c	15.2 ± 1.2d	8.9 ± 1e
		MLE	23.4 ± 1.42a	22.1 ± 1.3b	19.9 ± 1.3c	16.9 ± 1.27d	15 ± 1.2e
		GA	27.4 ± 1.8a	26.4 ± 1.5b	23.8 ± 1.4c	19.3 ± 1.2d	16.4 ± 1.1e
	SFW	Control	3.1 ± 0.2a	2.4 ± 0.21b	2.2 ± 0.18c	1.9 ± 0.17d	0.9 ± 0.08e
		MLE	3.8 ± 0.26a	3.1 ± 0.24b	2.9 ± 0.24b	2.5 ± 0.2c	1.7 ± 0.16d
		GA	6.1 ± 0.54a	5.4 ± 0.45b	5.1 ± 0.41c	5.0 ± 0.37c	3.3 ± 0.24d
	SDW	Control	2.1 ± 0.16a	1.5 ± 0.18b	1.1 ± 0.09c	0.9 ± 0.08d	0.4 ± 0.07e
		MLE	3 ± 0.22a	2 ± 0.2b	1.9 ± 0.22b	1.2 ± 0.16c	1.1 ± 0.17d
		GA	4.7 ± 0.33a	4.2 ± 0.29b	3.7 ± 0.28c	3.1 ± 0.27d	2.2 ± 0.26e
	RFW	Control	2.9 ± 0.29a	2 ± 0.19b	1.5 ± 0.11c	0.8 ± 0.04d	0.5 ± 0.04e
		MLE	3.4 ± 0.32a	2.9 ± 0.27b	2.2 ± 0.18c	1.6 ± 0.11d	1.2 ± 0.12e
		GA	5.7 ± 0.53a	4.9 ± 0.41b	4.3 ± 0.39c	3.7 ± 0.32d	3.1 ± 0.27e
	RDW	Control	1.9 ± 0.14a	1.6 ± 0.18b	1.3 ± 0.13c	0.8 ± 0.04d	0.6 ± 0.05e
		MLE	3.2 ± 0.3a	2.5 ± 0.31b	2 ± 0.29c	1.6 ± 0.26d	1.4 ± 0.18e
		GA	4.2 ± 0.43a	3.7 ± 0.35b	3 ± 0.3c	2.9 ± 0.29c	2.2 ± 0.16d

The data displayed are the means (± SE) of three replicates, and bars of dissimilar letters differ significantly at the *p* ≤ 0.05 level.

*MLE* Moringa leaf extract, *GA* green algae, *SL* shoot length, *RL* root length, *SFW* shoot fresh weight, *SDW* shoot dry weight, *RFW* root fresh weight, *RDW* root dry weight.

### Photosynthetic pigments

**Figure 1:**
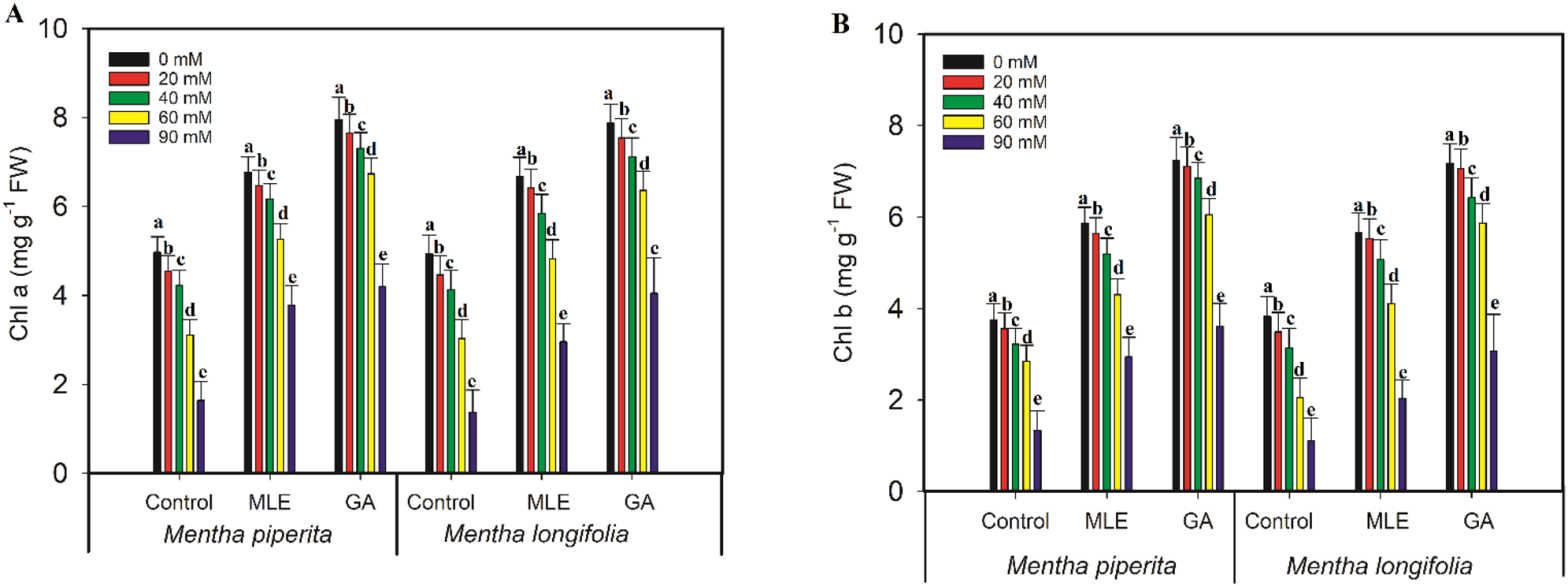
Effect of MLE (*Moringa oleifera*) and GA (*Ulva intestinalis*) on Chl a (**A**) and Chl b (**B**) in the *Mentha* species under salt stress (0 mM, 20 mM, 40 mM, 60 mM, 90 mM). The data displayed are the means (± SE) of three replicates, and bars of dissimilar letters differ significantly at the *p* ≤ 0.05 level.

In comparison to control seedlings, salt treatments (20, 40, 60, 90 mM) resulted in significant (*p* ≤ 0.001) decreases in total chlorophyll a and chlorophyll b content in *Mentha piperita* of 8%, 15%, 37%, 67% and 5%, 14%, 24%, 64% and in *Mentha longifolia* of 10%, 16%, 38%, 72% and 9%, 18%, 46%, 71%, respectively. Nonetheless, *Mentha* species treated with MLE and GA showed significantly greater chlorophyll content (*p* < 0.001) (Fig. 1).

### Proline content

In *Mentha piperita* and *Mentha longifolia*, the salt treatments (20, 40, 60, 90 mM) resulted to substantial (p < 0.001) increases in proline content of 9%, 19%, 30%, 42% and 9%, 21%, 32%, 40%, respectively, compared to those in control seedlings. When compared to the salt-stressed *Mentha* seedlings, the exogenous administration of MLE and GA considerably (p < 0.001) lowered proline content.

### Soluble sugar content

**Figure 2:**
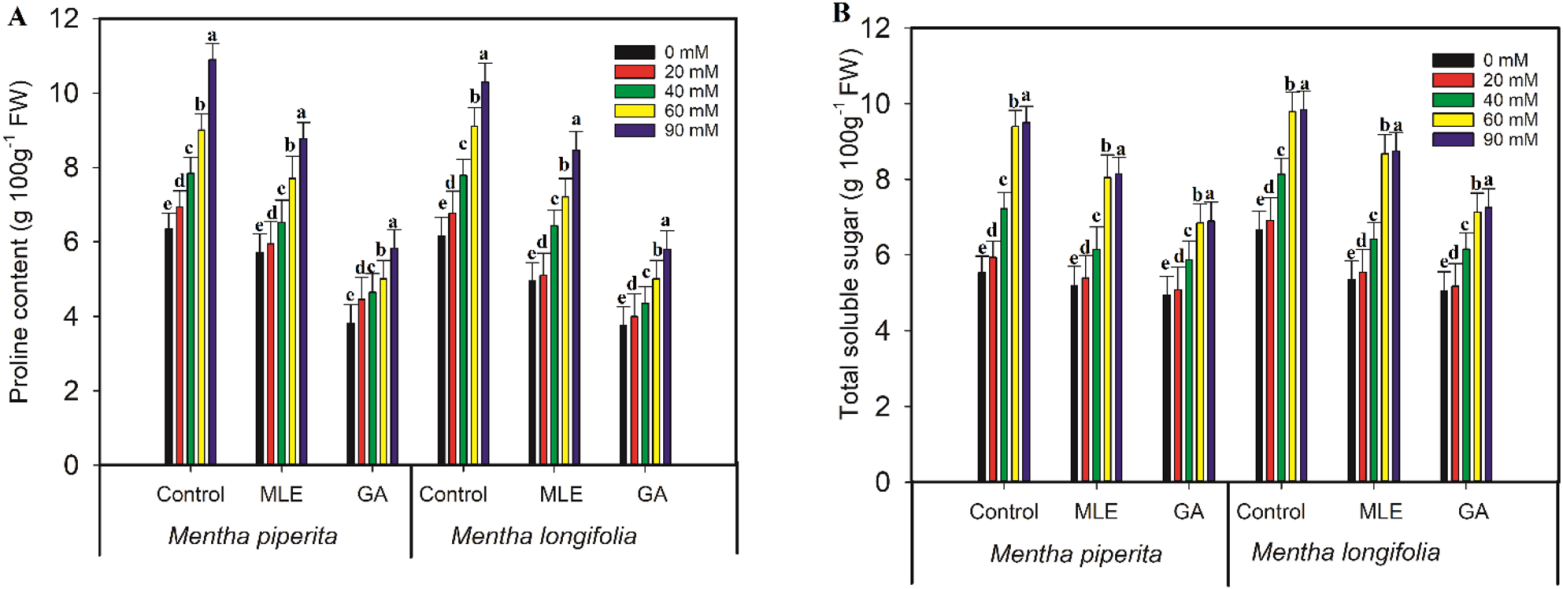
Effect of MLE (*Moringa oleifera*) and GA (*Ulva intestinalis*) on proline (**A**) and total soluble sugar (**B**) content in the *Mentha* species under salt stress (0 mM, 20 mM, 40 mM, 60 mM, 90 mM). The data displayed are the means (± SE) of three replicates, and bars of dissimilar letters differ significantly at the *p* ≤ 0.05 level.

Salt treatments of 20, 40, 60, and 90 mg/L raised the soluble sugar content in *Mentha piperita* by 6%; *Mentha longifolia* by 4%; *Mentha longifolia* by 18%; and *Mentha longifolia* by 32% compared to those in control seedlings that did not receive MLE and GE, respectively (Fig. 2). In spite of this, the exogenous infusion of MLE and GA considerably (p < 0.001) reduced the soluble sugar content.

### Antioxidant enzyme activity

**Figure 3:**
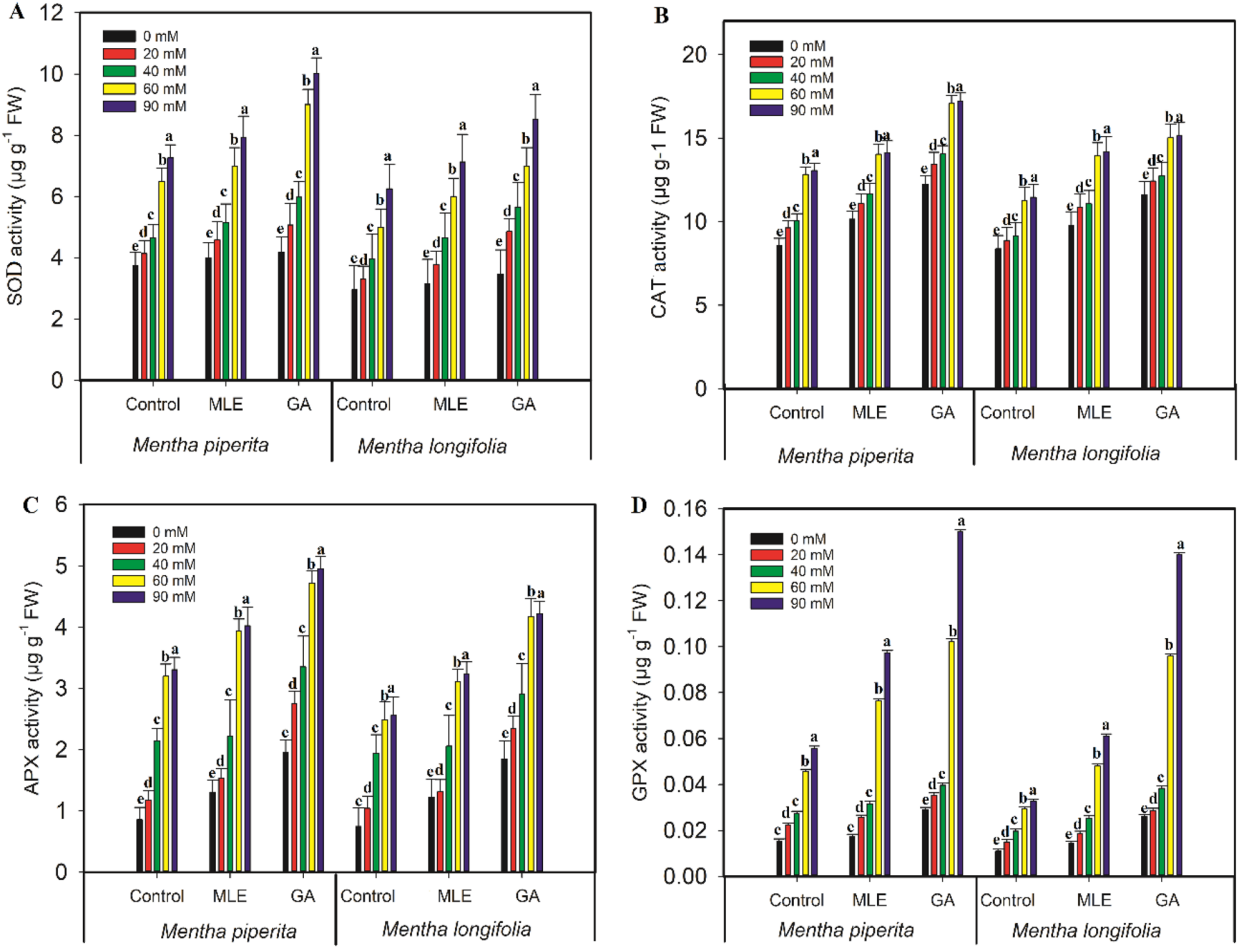
Effect of MLE (*Moringa oleifera*) and GA (*Ulva intestinalis*) on the antioxidant enzymes (**A** SOD; **B** CAT; **C** APX and **D** GPX) in the *Mentha* species under salt stress (0 mM, 20 mM, 40 mM, 60 mM, 90 mM). The data displayed are the means (± SE) of three replicates, and bars of dissimilar letters differ significantly at the *p* ≤ 0.05 level.

**Figure 4:**
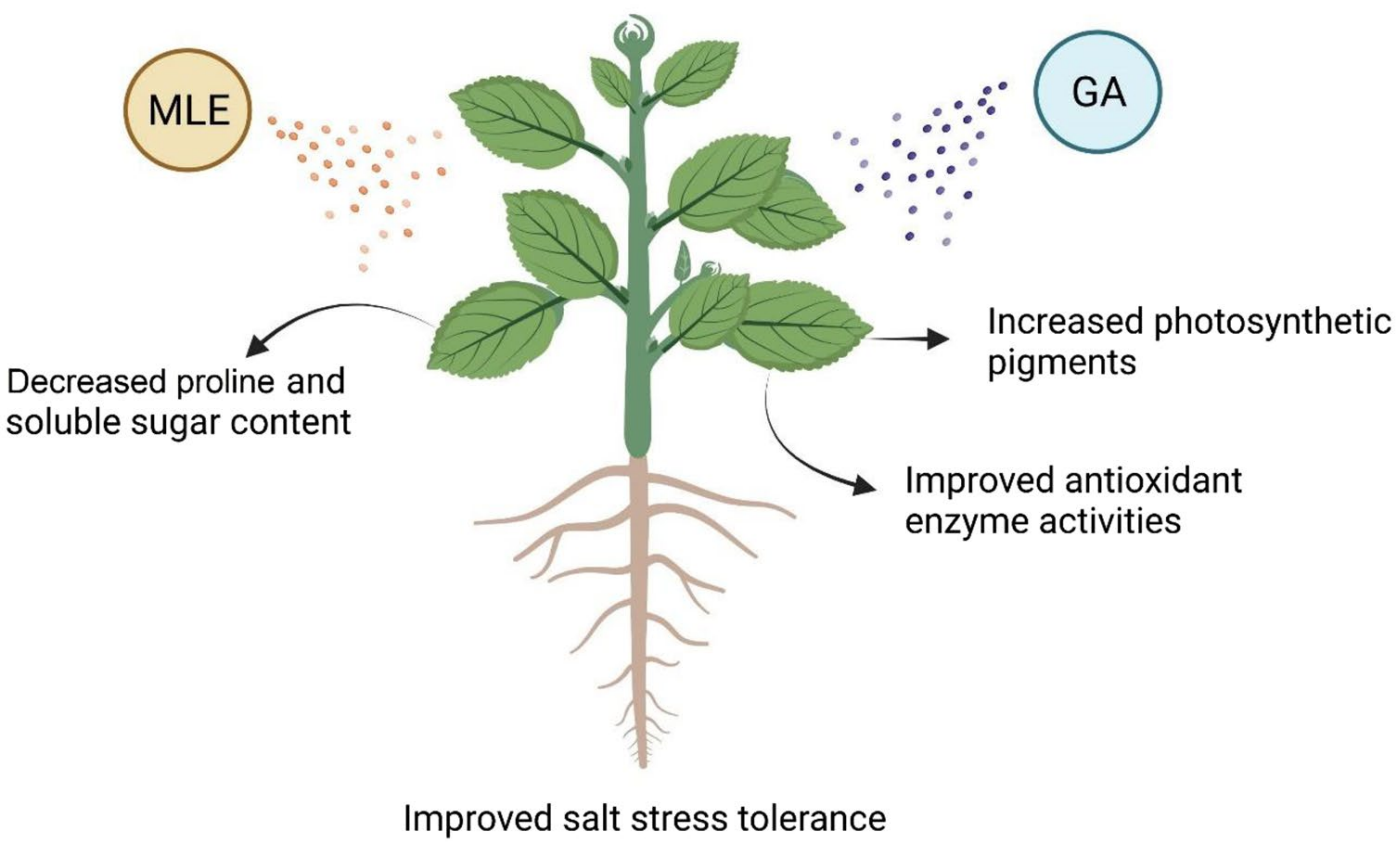
Schematic model figure shows how MLE and GA alleviates salinity stress in *Mentha* species.

20, 40, 60, and 90 mM salt treatments significantly (p ≤ 0.001) increased SOD activity by 10%, 19%,42%, and 49%, CAT activity by 11%, 15%, 33%, and 34%, APX activity by 27%, 60%, 73%, and 74%, GPX activity by 31%, 44%, 66% and 72%, respectively, relative to with respect to those in untreated *Mentha piperita* plants (Fig. 3). Correspondingly, the salt treatments (20, 40, 60, and 90 mM) led to significant (p ≤ 0.001) increases in SOD activity by 10%, 25%, 41%, and 53%, CAT activity by 6%, 9%, 26%, and 27%, APX activity by 28%, 62%, 70%, and 71%, GPX activity by 27%, 44%, 63%, and 66%, respectively, with respect to those in control *Mentha longifolia* seedlings. However, the application of MLE and GA resulted in a considerable improvement in these antioxidant enzyme parameters (Fig. 4). Furthermore, when exposed to high salt concentrations, GA treatment was more effective than MLE treatment in regulating these antioxidant enzymes (SOD, CAT, APX, and GPX).

## Discussion

According to the current report results, salt stress significantly reduced the shoot and root biomass of both *Mentha* seedlings. The decrease in growth caused by salinity could be attributed to decreased nutrient uptake by plants or increased sodium redistribution from roots to shoots^40^. However, the current study found that applying MLE and GA to *Mentha* species increased their growth and physiological greatly. Similar outcome was observed in rice where MLE increased the growth and biomass under drought stress^41^. These findings suggest that MLE and GA promote *Mentha* species growth by altering physiological processes.

In order to determine the level of salt stress, photosynthetic systems can be employed as indicators^42–44^. Reduced photosynthetic pigments are caused by salt stress and chlorophyll content was reported to be greater in stress-free conditions than in salt-stressed environments. In this present study, salt stress lowered the photosynthetic pigments of Mentha species. These findings back up the findings of Ahanger et al.^45^, who found that salt stress reduced chlorophyll concentration in wheat. In the current study, exogenous administration of MLE and GA significantly boosted the amount of photosynthetic pigments during salt stress. Moringa leaves are abundant in chlorophyll and carotenoids (xanthin, beta-carotene, alpha-carotene, and lutein), which have antioxidant effects^15^. MLE has also been shown to accelerate the synthesis of cytokinin’s, preventing early leaf senescence and resulting in a bigger leaf area with higher chlorophyll content^46^. The current study findings are consistent with Khan et al.^41^ discovery that MLE application significantly boosted photosynthetic pigments in wheat cultivated under favorable conditions. According to Yasmeen et al.^46^, foliar application of MLE during the tillering and heading phases increases chlorophyll a and b levels in wheat. The aqueous extract of *Ulva intestinalis* also increased the levels of chlorophyll a and b in parsley seedlings^47^.

The total soluble sugars and proline content were determined to understand more about MLE and GA effects on salt stressed seedlings. Total soluble sugars are well-known as one of the essential organic solutes that maintain cell homeostasis^48–50^, and proline aids in cell osmotic adjustment in the presence of salt stress^49,50^. According to our findings, total soluble sugars and proline levels increased in the *Mentha* species under salt stress when compared to the control condition. A similar study in chickpea found that salt stress boosted the synthesis of total soluble sugars and proline levels in wheat^49,51^. MLE and GA combined application reduced total soluble sugars and proline levels under salt stress. Seedlings of *Mentha* may be able to tolerate salt stress by lowering endogenous proline production. Similarly, when exposed to salt stress alone, MLE reduced the proline concentration in *Brassica napus* leaves^52^. Ibrahim et al.^53^ reported that ascorbic acid, betaine, glutathione, and proline are some of the bioactive components found in *Ulva lactuca* extract. These components, along with others, have the potential to alleviate the negative effects of salt stress.

Antioxidant defenses are essential in determining a plant's tolerance for stressful conditions^54–58^. With the beginning of salt stress, the activities of enzymatic antioxidants were found to be increased in the *Mentha* seedlings. Hanafy^59^ found a significant increase in the activities of enzyme antioxidants (GR, SOD, APX, and GPX) in rice that had been exposed to salt stress. The use of MLE and GA increased the antioxidant activity of enzymatic antioxidants in *Mentha* species, which was especially noticeable under salt stress. Increased SOD, CAT, APX, and GPX activity may be related with the activation of antioxidant responses that protect the plant from oxidative damage, according to our findings. According to Foyer and Noctor^60^, the initiation of enzymatic antioxidant activities in plants is a natural response for resisting oxidative stress. Similarly, MLE administration resulted in a significant increase in SOD activity in soybean, which was followed by the application of glutathione reductase (GR) and APX, respectively. Zaki and Rady^61^ found that seed soaking or foliar spray treatment of MLE increased the antioxidant enzyme activities such as SOD, and APX in common bean (*Phaseolus vulgaris* L.) plants. Microalgae, on the other hand, were found to boost SOD, CAT, APX, and peroxidase (POD) activities in wheat seedlings under salt stress^62^. Furthermore, similar studies were conducted on several plants and showed that using *Ulva lactuca* and marine algae extracts increased the antioxidant enzyme activities. The increase in enzyme activity could be indicative of the presence of antioxidant and osmoprotectant substances.

## Conclusion

Salt stress has a deleterious impact on the growth and physiology of the *Mentha* species. MLE and GA demonstrated the best biostimulant potential in terms of improved growth and physiology of *Mentha* seedlings grown under normal and salt stress. Foliar application of MLE and GA significantly improved photosynthetic pigments, osmolytes, and antioxidant enzyme activity under normal and salt stress conditions. Overall, these findings suggest that MLE and GA can be used to promote field plant development in both normal and salt-stressed environments. More research is required, however, to determine the effectiveness of MLE and GA in reducing the harmful effects of soil salinization on plants, as well as the optimal dose. Furthermore, the molecular processes underlying MLE and GA-mediated salt tolerance in plants must be understood.
